# Effects of sports experience on children's gross motor coordination level

**DOI:** 10.3389/fspor.2023.1310074

**Published:** 2023-12-22

**Authors:** Valentina Biino, Valerio Giustino, Maria Chiara Gallotta, Marianna Bellafiore, Giuseppe Battaglia, Massimo Lanza, Carlo Baldari, Matteo Giuriato, Flavia Figlioli, Laura Guidetti, Federico Schena

**Affiliations:** ^1^ Department of Human Sciences, Università degli Studi di Verona, Verona, Italy; ^2^ Department of Neurosciences, Biomedicine and Movement Sciences, Università degli Studi di Verona, Verona, Italy; ^3^ Sport and Exercise Sciences Research Unit, Department of Psychology, Educational Science and Human Movement, Università degli Studi di Palermo, Palermo, Italy; ^4^ Department of Physiology and Pharmacology “Vittorio Erspamer”, Sapienza Università di Roma, Roma, Italy; ^5^ Department of Theoretical and Applied Sciences, Università Telematica eCampus, Novedrate, Italy; ^6^ Department of Humanities, Movement, and Education Sciences, Università Telematica degli Studi Niccolò Cusano, Roma, Italy

**Keywords:** sports practice, gymnastics, cycling, athletics, swimming, BMI, physical activity level

## Abstract

**Background:**

Gross motor coordination (GMC) development could be influenced by age, gender, weight status, geographical area, living setting, home environment, socio-economic status, sports practice.

**Purpose:**

To verify whether practicing sports and practicing different sports could influence children's GMC level.

**Methods:**

A total of 295 children aged 8–11 years were involved in the study and divided into 5 groups in relation to the sport they practiced: gymnastics group (*n* = 67; 51F, 16M), cycling group (*n* = 64; 15F, 49M), athletics group (*n* = 47; 22F, 25M), swimming group (*n* = 35; 20F, 15M), control group (*n* = 82; 42F, 40M). The four subtests of the Körperkoordinations Test für Kinder (KTK) assessed children's GMC level. The scores from each of the four subtests were summed into the KTK total raw score (RS) and then converted into a gender- and age-specific motor quotient (MQ).

**Results:**

Children practicing sports showed significantly higher RS and MQ score than children of control group (203.14 ± 38.55 vs. 163.63 ± 43.50 and 98.56 ± 15.79 vs. 83.01 ± 16.71, respectively; *p* < 0.001). Children practicing gymnastics had a significantly higher RS and MQ than children of cycling, swimming, and control groups (*p* < 0.05), children of control group had a significantly lower RS and MQ than children of all other groups (*p* < 0.05). Children practicing gymnastics performed better walking backwards subtest than all other children's groups (*p* < 0.001). Children of control group performed worse jumping sideways subtest than children of gymnastics, athletics and swimming groups (*p* < 0.01). Children practicing gymnastics performed better moving sideways subtest than children of athletics, cycling and control groups (*p* < 0.01); children of control group performed worse than children of all other groups (*p* < 0.01). Children of control group performed worse hopping for height subtest than children of gymnastics, athletics and cycling groups (*p* < 0.05); children practicing gymnastics performed better than children of swimming and control groups (*p* < 0.05).

**Conclusions:**

The performance model and therefore the specialized training that each sport discipline required, could justified the differences in children's GMC level among sports groups. Thus, coaches should plan individualized interventions and choose activity contents to support children's GMC development.

## 1. Introduction

Motor coordination refers to the level of ability to perform a wide range of motor tasks, including fundamental movement skills (FMS) (1, 2). These are defined as the building blocks for the execution of more complex movements required for the practice of sporting activities across lifespan (3). FMS can be divided into fine and gross motor skills and include object control skills (e.g., throwing, catching, and kicking), locomotor skills (e.g., running, jumping, and hopping), and balance skills (e.g., balancing, one-foot balance, and swinging) (3, 4). Based on the predictability of environmental changes, motor skills are divided into open skills and closed skills (5). Increasing the unpredictability of the environmental context, adding variable movement structures, and increasing interpersonal interaction processes can increase cognitive challenges (6, 7).

Motor coordination includes the ability to adapt movement patterns and adjust the forces to complete a task successfully (8). Adapting motor skills or creating new solutions to successfully complete a task should be the first goal when learning the basics of motor coordination. According to Bernstein, motor coordination consists in overcoming excessive degrees of freedom of our motor organs, that is transforming the motor organs into controllable systems, but the difficult of motor control lies in the change of the environment which requires solving unexpected problems (9). For this reason, “motor wits” should be exercised (10).

Several factors could influence motor coordination development, such as age, gender, and weight status (11), geographical area and living setting (12), home environment and socio-economic status (13, 14). Motor coordination development during childhood plays a crucial role for the engagement in sporting activities that are precisely refined with sports practice (15, 16). Therefore, an adequate level of motor coordination during development allows children to carry out the activities of daily living and favour their participation and success in sporting activities (17). Although the practice of sporting activities seems to positively influence motor coordination, this relationship has not been thoroughly investigated. Indeed, very few studies explored the potential influence of practice of different sports on motor coordination development (18–24). Several test batteries were developed over time to evaluate children's motor development and to provide a measure of specific motor skill performance useful for young talents identification in sports (25, 26).

Hence, existing studies investigated the levels of motor coordination in children and adolescents practicing a single type of sporting activity and the differences of motor coordination according to age, gender, and level of players (22, 23). Previous studies investigated the differences of motor coordination among children and adolescents practicing different sporting activities (18–20). Finally, the effects of a sport-specific curriculum or training model on motor coordination were investigated (21, 24). Rudd et al. (21) revealed the effectiveness of a gymnastics training programme at developing children's stability skills and object control skills without hindering the development of their general body coordination and locomotor skills. Moreover, Trajković et al. (24) showed the effectiveness of a neuromuscular volleyball training on adolescent's motor competence.

Therefore, the first aim of the present study was to verify whether practicing sports could influence children's gross motor coordination level. Specifically, we compared the gross motor coordination level of children practicing sports with that of children who did not practice any sport. The second aim was to verify whether practicing different sports could influence children's gross motor coordination level differently. Specifically, we investigated whether the practice of four different closed-skills sports (athletics, swimming, gymnastics, and cycling) could differently influence the development of children's motor coordination. We hypothesized that sport experience allows children to achieve a suitable level of motor coordination and that the amount of improvement in motor coordination could depend on the type of sporting activity practiced.

## 2. Materials and methods

### 2.1. Participants

Two hundred and ninety-five children (139 f, 133 m; mean age: 9.6 ± 0.8 years; weight: 33.6 ± 7.8 kg; height: 137.6 ± 8.2 cm; BMI: 17.6 ± 2.9 kg/m^2^) took part in the study. Participants were recruited through convenience sampling, that is, parents, who enrolled their children in the sports centres, chose the sport course based on their own and their children's preference.

Participants were divided into five groups: four competitive sports groups and one control group. The first sport group was composed of 47 children (22 f, 25 m; mean age: 9.7 ± 0.9 years; weight: 33.9 ± 7.8 kg; height: 139.7 ± 9.9 cm; BMI: 17.2 ± 2.1 kg/m^2^), who performed athletics. The second sport group was composed of 35 children (20 f, 15 m; mean age: 9.5 ± 0.9 years; weight: 32.1 ± 7.2 kg; height: 136.2 ± 8.6 cm; BMI: 17.2 ± 2.4 kg/m^2^), who practiced swimming. The third and fourth groups consisted of 67 (51 f, 16 m; mean age: 9.7 ± 0.8 years; weight: 31.4 ± 6.9 kg; height: 136.6 ± 9.1 cm; BMI: 16.7 ± 2.0 kg/m^2^) and 64 (15 f, 49 m; mean age: 9.6 ± 1.0 years; weight: 34.5 ± 6.7 kg; height: 136.8 ± 8.0 cm; BMI: 18.4 ± 2.7 kg/m^2^) children who practiced gymnastics and cycling respectively. For more details, see the Supplementary Appendix. The control group consisted of 82 children (42 f, 40 m; mean age: 9.5 ± 0.4 years; weight: 34.8 ± 8.9 kg; height: 138.3 ± 6.4 cm; BMI: 18.1 ± 3.8 kg/m^2^) recruited from primary school and not involved in any after-school structured sport course.

The inclusion criteria were: be aged between 8 and 10 years; having at least two years of sport practice (for children belonging to sports groups); not attending any structured sports activity (for children of the control group).

All children of the sports groups had two consecutive years of sport practice in the specific sport disciplines who they belonged at the time of measurements. All the measurements of this study were conducted from November 2022 to May 2023 and carried out by physical education specialists.

Written informed consent was obtained from parents (or legal guardians) prior to study participation.

### 2.2. Anthropometric measurements

Participant's height was measured with a stadiometer to the nearest 0.5 cm. Weight was measured using a scale to the nearest 0.1 kg. Body mass index (BMI) was calculated by dividing the weight in kilograms by the square of the height in meters (kg/m^2^).

### 2.3. Gross motor coordination measurement

The assessment of children's gross motor coordination consisted of the four standardized subtests of the Körperkoordinations Test für Kinder (KTK): jumping sideways, hopping for height, walking backwards on balance beams, moving sideways (27, 28).

The following two subtests assess the strength component of gross motor coordination:

*Jumping sideways*—children had to jump sideways over a board (60 cm × 4 cm × 2 cm) as many times as possible in 15 s for two trials. The number of jumps was summed over the two trials.
*Hopping for height*—children had to hop on one leg over an increasing pile of pillows (5 cm each) after a short run-up. Three, two, or one point(s) were awarded for successful performance in the first, second, or third trial, respectively. A maximum of 39 points (ground level plus 12 pillows) could be scored for each leg, yielding a possible maximum score of 78.

The following two subtests assess the balance and dexterity components of gross motor coordination:

*Walking backwards on balance beams*—children had to walk backwards on three different balance beams of decreasing width (6, 4.5, and 3 cm, respectively), over three trials for beam. A maximum of 24 steps (eight per trial) was counted for each balance beam, for a maximum of 72 steps.
*Moving sideways*—children had to move sideways as quickly as possible over two boards (25 cm × 25 cm × 5.7 cm) in 20 s. The number of transfers was counted and summed over two trials.

The raw scores from each of the four subtests were recorded and summed into the KTK total raw score (RS). Furthermore, the RSs from each of the four subtests were converted into gender- and age-specific motor quotients (MQ) values based on the performance of 1,228 typically developing German children. The scoring of the KTK test was performed according to the guidebook (27, 28). The total MQ was then calculated by adding the four MQ scores. The total MQ defines the level of gross motor coordination, and values between 86 and 115 describe the normality (27, 28).

The test–retest reliability coefficient for the raw score on the total test battery was reported as 0.97, while corresponding coefficients for individual tests ranged from 0.80 to 0.96. Both factor analysis and inter-correlations indicated acceptable construct validity (28–31).

### 2.4. Physical activity level evaluation

Children's physical activity level was assessed using the Italian version of the Physical Activity Questionnaire for Older Children (PAQ-C-It) (32). It is a 7-day-recall self-administered instrument and consists of nine items related to sports, physical activities at school, and leisure-time activities, including the weekend. Responses were based on a five-point scale (ranging from 1 to 5). The final score was calculated by averaging the scores from all the questions.

### 2.5. Statistical analysis

Data were described by means and standard deviations.

#### 2.5.1. Differences between children practicing sports and children of the control group

The unpaired comparison *t*-test was performed to verify differences on RS and MQ scores, BMI (kg/m^2^), and physical activity levels (scores) between children practicing sports and children of the control group.

#### 2.5.2. Differences among groups on RS and MQ scores, BMI, and physical activity levels

ANOVA was performed to examine differences and interactions on RS and MQ scores, BMI (kg/m^2^), and physical activity levels (scores) between boys and girls from different groups (athletics vs. swimming vs. gymnastics vs. cycling vs. control group). The analyses were followed by *post hoc* analysis (Bonferroni adjustment) when significant main effects or interactions were observed. Effect size was also calculated using Cohen's definition of small, medium, and large effect size (as partial *η*^2^ = 0.01, 0.06, 0.14, respectively) (33).

#### 2.5.3. Differences among groups on KTK subtests scores

ANOVA was performed to examine differences and interactions on KTK subtests raw and adjusted scores between boys and girls from different groups (athletics vs. swimming vs. gymnastics vs. cycling vs. control group). The analyses were followed by *post hoc* analysis (Bonferroni adjustment) when significant main effects or interactions were observed. Effect size was also calculated using Cohen's definition of small, medium, and large effect size (as partial *η*^2^ = 0.01, 0.06, 0.14, respectively) (33).

#### 2.5.4. Association between children's MQ, BMI, and physical activity level

Correlation analysis was used to explore the relationships between RS and MQ scores with BMI and physical activity levels. A multiple linear stepwise regression analysis was then performed to examine the associations of MQ scores with correlated variables.

Statistical significance was set at *p* ≤ 0.05, and all analyses were performed using IBM SPSS statistics version 27.

## 3. Results

### 3.1. Differences between children practicing sports and children of the control group

Children practicing sports showed significantly higher RS and MQ scores and physical activity levels (PAQ-C-It score) than children of control group (Table 1).

**Table 1:** KTK total raw score (RS), motor quotient (MQ), body mass index (BMI), and physical activity level (PAQ-C-It) of children practicing sports and children of the control group.

	Sports group	Control group
RS (score)	203.14 ± 38.55	163.63 ± 43.50*
MQ (score)	98.56 ± 15.79	83.01 ± 16.71*
BMI (kg/m^2^)	17.37 ± 2.40	18.10 ± 3.82
PAQ-C-It (score)	2.62 ± 0.45	1.96 ± 0.61*

* *p* < 0.001 Control group vs. Sports group.

### 3.2. Differences among groups on RS and MQ scores, BMI, and physical activity levels

**Figure 1:**
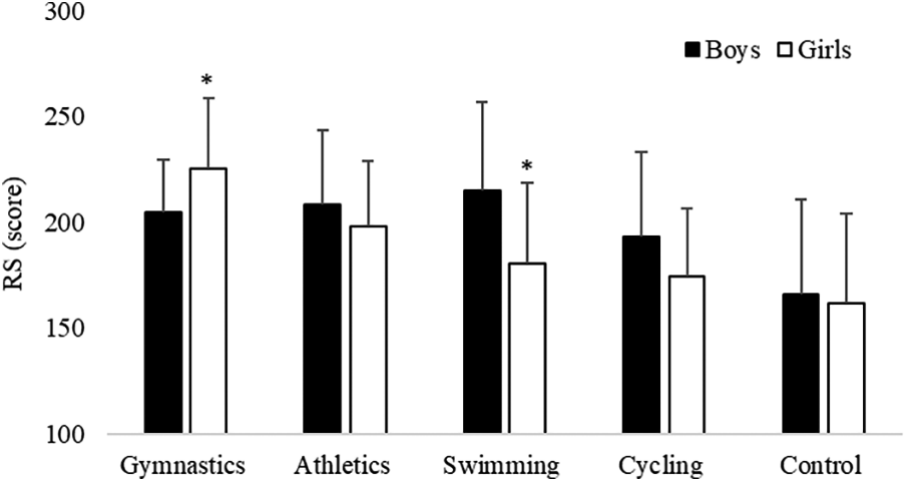
RS of boys and girls practicing gymnastics, athletics, swimming, cycling and of the control group (*p = 0.02).

Differences for group (*F*_4,285 _= 17.22, *p* < 0.001, large effect size *η*^2^ = 0.20) revealed that children practicing gymnastics had a significantly higher RS than children of cycling, swimming, and control groups, while children of control group had a significantly lower RS than children of all other groups (Table 2). Moreover, group × gender interaction (*F*_4,285 _= 3.05, *p* = 0.02, small effect size *η*^2^ = 0.04) showed that girls of gymnastics group had a higher RS than boys while boys of swimming group had a higher RS than girls (Figure 1).

**Table 2:** KTK total raw score (RS), motor quotient (MQ), body mass index (BMI), and physical activity level (PAQ-C-It) of children practicing gymnastics, athletics, swimming, cycling and children of the control group.

	Gymnastics group	Athletics group	Swimming group	Cycling group	Control group
RS (score)	220.67 ± 32.28^‡,§,‖^	203.55 ± 33.19^‖^	195.26 ± 43.10^*,‖^	188.80 ± 39.12^*,‖^	163.63 ± 43.50^*,†,‡,§^
MQ (score)	105.45 ± 14.50^‡,§,‖^	98.06 ± 13.99^‖^	95.17 ± 16.36^*,‖^	93.58 ± 15.77^*,‖^	83.01 ± 16.71^*,†,‡,§^
BMI (kg/m^2^)	16.65 ± 2.02^§,‖^	17.16 ± 2.09	17.18 ± 2.42	18.38 ± 2.69*	18.10 ± 3.82*
PAQ-C-It (score)	2.57 ± 0.49^‡,‖^	2.49 ± 0.48^‡,‖^	2.94 ± 0.32^*,†,§,‖^	2.61 ± 0.38^‡,‖^	1.96 ± 0.61^*,†,‡,§^

* *p* < 0.01 vs. Gymnastics. ^†^*p* < 0.01 vs. Athletics. ^‡^*p* < 0.05 vs. Swimming. ^§^*p* < 0.01 vs. Cycling. ^||^*p* < 0.01 vs. Control.

Differences for group (*F*_4,285 _= 22.07, *p* < 0.001, large effect size *η*^2^ = 0.24) revealed that children practicing gymnastics had a higher MQ than children of cycling, swimming and control groups, while children of control group had a lower MQ than children of all other groups (Table 2). Differences for gender (*F*_1,285 _=_ _9.08, *p* = 0.003, small effect size *η*^2^ = 0.03) revealed that boys had higher MQ than girls (95.61 ± 17.31 vs. 92.91 ± 17.59 scores, respectively).

Moreover, differences for group (*F*_4,262 _= 4.60, *p* = 0.001, medium effect size *η*^2^ = 0.07) revealed that children practicing gymnastics had a lower BMI than children of cycling and control groups (Table 2).

Differences for group (*F*_4,262 _= 32.22, *p* < 0.001, large effect size *η*^2^ = 0.33) revealed that children practicing swimming had a higher level of physical activity (PAQ-C-It score) than children of all other groups, while children of control group had a lower level of physical activity than children of all other groups (Table 2). Moreover, differences for gender (*F*_1,262 _=_ _4.94, *p* = 0.03, small effect size *η*^2^ = 0.02) revealed that boys had higher physical activity levels than girls (2.47 ± 0.60 vs. 2.38 ± 0.57 scores, respectively).

### 3.3. Differences among groups on KTK subtests scores

#### 3.3.1. KTK subtests raw scores

**Figure 2:**
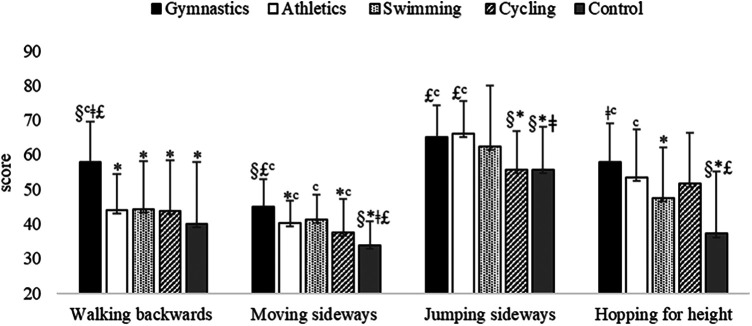
KTK subtests raw scores of children practicing gymnastics, athletics, swimming, cycling and children of the control group. *p < 0.01 vs. Gymnastics; ^§^*p* < 0.01 vs. Athletics; ^‡^*p* < 0.05 vs. Swimming; £*p* < 0.01 vs. Cycling; ^c^*p* < 0.01 vs. Control group.

Differences for group (*F*_4,285 _= 11.52, *p* < 0.001, large effect size *η*^2^ = 0.14) revealed that children practicing gymnastics performed better walking backwards subtest than all other children's groups (Figure 2).

Differences for group (*F*_4,285 _= 12.39, *p* < 0.001, large effect size *η*^2^ = 0.15) revealed that children of control group performed worse jumping sideways subtest than children of gymnastics, athletics and swimming groups. Moreover, children of cycling group performed worse than children of gymnastics and athletics groups (Figure 2). Differences for gender (*F*_1,285 _= 13.25, *p* < 0.001, small effect size *η*^2^ = 0.04) revealed that boys performed better than girls (61.26 ± 13.00 vs. 59.32 ± 12.39 scores, respectively).

Differences for group (*F*_4,285 _= 21.07, *p* < 0.001, large effect size *η*^2^ = 0.23) revealed that children practicing gymnastics performed better moving sideways subtest than children of athletics, cycling and control groups. Moreover, children of control group performed worse than children of all other groups (Figure 2). Differences for gender (*F*_1,285 _=_ _6.75, *p* = 0.01, small effect size *η*^2^ = 0.02) revealed that boys performed better than girls (39.24 ± 8.81 vs. 38.94 ± 8.84 scores, respectively).

Differences for group (*F*_4,267 _= 13.05, *p* < 0.001, large effect size *η*^2^ = 0.16) revealed that children practicing gymnastics performed better hopping for height subtest than children of swimming and control groups. Moreover, children of control group performed worse than children of gymnastics, athletics and cycling groups (Figure 2). Differences for gender (*F*_1,267 _=_ _12.24, *p* = 0.001, medium effect size *η*^2^ = 0.04) revealed that boys performed better than girls (51.33 ± 16.90 vs. 48.47 ± 15.65 scores, respectively).

#### 3.3.2. KTK subtests adjusted scores

**Figure 3:**
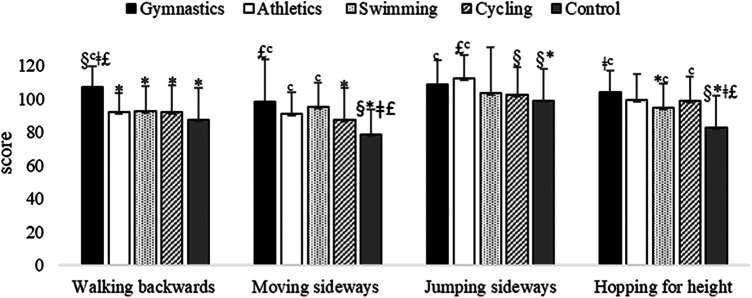
KTK subtests adjusted scores of children practicing gymnastics, athletics, swimming, cycling and children of the control group. *p < 0.01 vs. Gymnastics; ^§^*p* < 0.01 vs. Athletics; ^‡^*p* < 0.05 vs. Swimming; £*p* < 0.01 vs. Cycling; ^c^*p* < 0.01 vs. Control group.

Differences for group (*F*_4,285 _= 12.09, *p* < 0.001, large effect size *η*^2^ = 0.15) revealed that children practicing gymnastics performed better walking backwards subtest than all other children's groups (Figure 3).

Differences for group (*F*_4,285 _= 8.33, *p* < 0.001, medium effect size *η*^2^ = 0.11) revealed that children of control group performed worse jumping sideways subtest than children of gymnastics and athletics groups. Moreover, children practicing cycling performed worse than children practicing athletics (Figure 3). Differences for gender (*F*_1,285 _=_ _33.86, *p* < 0.001, medium effect size *η*^2^ = 0.11) revealed that boys performed better than girls (109.77 ± 15.89 vs. 99.51 ± 19.65 scores, respectively).

Differences for group (*F*_4,285 _= 15.08, <0.001, large effect size *η*^2^ = 0.18) revealed that children practicing gymnastics performed better moving sideways subtest than children of cycling and control groups. Moreover, children of control group performed worse than children of all other groups (Figure 3). Differences for gender (*F*_1,285 _=_ _4.34, *p* = 0.04, small effect size *η*^2^ = 0.02) revealed that boys performed better than girls (89.61 ± 18.25 vs. 88.09 ± 21.11 scores, respectively).

Differences for group (*F*_4,285 _= 17.02, <0.001, large effect size *η*^2^ = 0.19) revealed that children practicing gymnastics performed better hopping for height subtest than children of swimming and control groups. Moreover, children of control group performed worse than children of all other groups (Figure 3). Differences for gender (*F*_1,285 _=_ _4.59, *p* = 0.03, small effect size *η*^2^ = 0.02) revealed that boys performed better than girls (96.41 ± 17.35 vs. 93.64 ± 18.21 scores, respectively).

### 3.4. Association between children's MQ, BMI, and physical activity level

Children's RS and MQ scores negatively correlated with BMI and positively correlated with physical activity level (Table 3).

**Table 3:** Correlation coefficients between RS and MQ scores with BMI and physical activity levels.

	RS	MQ
BMI (kg/m^2^)	−0.321*	−0.359*
Physical activity level (score)	0.451*	0.457*

* *p* < 0.01.

The application of the multiple regression analysis indicates that physical activity level and BMI predicted MQ score, although the percentage of variance was moderate (*R*^2^ = 33%) (Table 4).

**Table 4:** Stepwise linear regression model to predict MQ.

	Predictor variables	SE	Adjusted *R*^2^	*R* ^2^	Change in *R*^2^	*p*	Constant	*β*
Model 1		15.143	0.238	0.240	0.240	0.000	59.331	
	Physical activity level (score)							0.490
Model 2		14.291	0.321	0.326	0.086	0.000	95.105	0.425 −0.300
	Physical activity level (score) BMI (kg/m^2^)							

## 4. Discussion

The aim of the present study was to verify whether the practice of sports could influence the level of gross motor coordination in children. A further aim was to verify whether the practice of different sports could influence children's gross motor coordination level differently. Our hypothesis was that children could achieve different levels of motor coordination depending on the type of sporting activity practiced.

### 4.1. Can practicing sports influence motor coordination?

The results of our study showed that children who practiced sports showed significantly higher levels of motor coordination and physical activity than children in the control group (Table 1). Moreover, MQ was negatively correlated with BMI and positively correlated with physical activity level (Tables 3, 4), in line with previous studies (34, 35).

Although few, previous research has investigated the role of sports practice on motor coordination, also considering possible influencing factors. In particular (22), investigated the motor coordination level of young tennis players in relation to age and gender. Authors found that none of the players showed a motor coordination level lower than the normal level and that about 40% of the players showed a motor coordination level higher than the normal level. Moreover, no gender differences on motor coordination were detected (22). In a more recent study, Söğüt et al. reported a significantly higher motor coordination performance in young elite tennis players than club level tennis players (23). Similarly, a recent study, exploring motor coordination level among young soccer players and any age-related differences, showed that none of the players showed a motor coordination level lower than the normal level (36). Moreover, authors found that about the 10% of the players showed a motor coordination level higher than the normal level. Although no significant age-related differences were found on MQ, older players reported higher raw scores in all subtests of the coordinative battery used than younger players.

The results of the jumping subtest showed a significant difference between boys and girls who practice sports and those in the control group (mean score: male 56.07 ± 12.76; female 48.24 ± 12.19; vs. control group male 37.38 ± 19.49; female 37 ± 6.89). This subtest assesses a fundamental motor skill, i.e., jumping through the “hopping for height” task, the monitoring of which should not be underestimated. Indeed, some previous studies demonstrated that the most significant models on the use of the KTK test are never those that present a reduced version, that is, without the “hopping for height” subtest (37) The control group of our study showed significantly lower values in jumping motor ability, both in male and female children, compared to children who practiced sports. The MQ of the control group was not only significantly lower than children who practiced sports, but it did not even reach the normal level, which corresponds to the score range between 85 and 115 (27, 28).

In agreement with Hudson and Willoughby, FMS should be learned already in early childhood, as they promote children's physical, cognitive, and social development (38). Learning FMS creates the conditions for fine and gross motor coordination (9). Motor skills play an important role throughout life (39, 40). Control of motor skills influences engagement and persistence in physical activity (41–43). Being able to jump, run, throw, catch interacts positively with participation in physical activity and sport, with a benefit for the physical health of both children and adolescents (16, 44). We found that children in the control group had significantly lower physical activity levels than children in the sports group (Table 1). This result is in agreement with the study by Barnett and colleagues who define the practice of physical activity as one of the determining factors of motor coordination (1). In a recent study on individuals of both sexes between the ages of 6 and 19, Coppens and colleagues highlighted that, regardless of gender, age, and BMI level, individuals who participate in sports show higher MQ values than to those who do not practice sports (45). This means that by considering the entire sample, regardless of age, gender, and BMI, and dividing them only on the basis of sports practice (yes/no), those who practiced sports had higher MQ values than those who did not practice it. Our results agree with those existing in the literature. In fact, we can affirm that practicing sport predicts better levels of motor coordination and BMI than not practicing it.

### 4.2. Can practicing different types of sports have different effects on motor coordination?

Motor development should be addressed through appropriately designed sports programs (46). This study examined the effectiveness of different sports interventions on the gross motor coordination in children. As already stated, some studies have been mentioned regarding the positive effects of sports practice on motor development of school-age children (18–24, 36, 45). However, it has never been defined which type of sport shows the greatest benefits on motor coordination. Marinkovic et al. found a significant higher MQ in young female dancers when compared to a control group consisting of girls practicing other sporting activities (19). Jaakkola et al, comparing motor coordination performances among adolescent athletes practicing gymnastics, swimming, and ice hockey, detected a significant higher MQ in gymnasts compared to ice hockey players (18). Moreover, Popović et al, found significant higher levels of MQ in children enrolled in multisport activities than children engaged in soccer (20). Another study on this topic showed the effectiveness of a 16-week gymnastics curriculum at developing children's balance and object control skills when compared to a standard physical education curriculum (21).

Our results revealed that children who practice gymnastics had a higher MQ than children who practice cycling and swimming (Table 2). It seems that practicing gymnastics leads to a higher level of motor coordination than practicing other sports in which the continuous and cyclical repetition of a specific movement is emphasized.

We speculate that the results obtained by children practicing gymnastics were favored by the fact that the backward walking subtest of the KTK test is a typical skill trained in this sport. Therefore, although one might think that some motor coordination assessment tools could give greater advantages to some sports compared to others, in reality this is only apparently possible. In fact, riding a bicycle and backwards on a board have elements in common because they both require maintaining a dynamic balance. These seemingly different abilities represent similar forms of motion transfer.

Motor coordination depends on the human ability to control large muscle masses synergistically, organize the temporal course of movements by controlling their rhythmic execution, and providing alternating activity of flexor and extensor muscles (9). It is closely related to the development of motor skills and the automation of movements, and it is designed to provide the internal consistency of a movement in order to coordinate the actions of all muscles. However, the acquisition of motor skills and their automatisms could not to be sufficient to adapt such a complex and balanced movement to changes in external conditions of the real environment (7, 47, 48).

Teachers and coaches should choose exercises and environments to create tasks and environmental constrains to stimulate the development of greater sensory-cognitive skills related to the specific sport domain (49). This should also be proposed for the acquisition of skills characterized by the repetition of a cyclical movement such as pedaling or running.

## 5. Conclusion

Our results showed that already at the age of 8–10 years, structured sporting activity has a specific impact on motor coordination development. In fact, children presented an early specialization in motor coordination based on the sport practiced. These differences in gross motor coordination level among different sports disciplines could be associated with the sport-specific performance model and training. Although the technical guides of sports federations regarding the education of school-age children do not seem to neglect the aspect of multidirectional work, the different types of sport practiced would seem to lead to motor diversification. In fact, the sample we recruited showed that the specificity of a sport acts differently on motor coordination. In this period of development, intentional or deliberate play, characterized by enjoyment and immediate rewards, should be emphasized (50). Deliberate play involves a set of implicit or explicit rules that children or adults can often change. The ability to play, explore and experiment in various movement situations is crucial for the development of fundamental motor skills (50). Thus, coaches should plan individualized interventions and choose multidirectional activity contents to support children's motor coordination development.

### 5.1. Strengths and limitations

This study is not without limitations. Unbalanced sports groups in numerical terms limit the representativeness and therefore the generalizability of our results. Another limitation is the lack of randomized participant recruitment. Moreover, the study investigated the possible different influence that the practice of only four different closed-skills sports (athletics, swimming, gymnastics, and cycling) could have on of children's motor coordination development. Despite these limitations, the present study provided evidence on the impact that different types of sport might have on motor coordination.

Future studies are needed to investigate many different sports influences on motor coordination. Moreover, it would be interesting to study situation sports, also called sports games such as soccer and basketball, in the near future. We did not consider ball sports in this study because we expected to find fewer differences and the risk was that the stimulations would be more contrasting. However, it would be interesting to compare the results provided by sports in this study with situational ones.

## Data Availability

The raw data supporting the conclusions of this article will be made available by the authors, without undue reservation.
