# Supplementary material for: Effects of sports experience on children's gross motor coordination level
Source: Front Sports Act Living. 2023 Dec 22;5:1310074. doi: 10.3389/fspor.2023.1310074 (PMC10766855; doi:10.3389/fspor.2023.1310074)
Supplement: Supplementary file 1 [file Table1.docx]

**Appendix**

*Gymnastics group training*

Analyzing the technical guide of the Italian Gymnastics Federation, in the sector that examines basic motor activities, elements such as the upside down, the handstand, the wheel are considered transferable learning. Therefore, the teaching of these elements is not based on the technical quality of their execution, but on the enrichment of motor experiences which will be developed and consolidated only later to encourage purely technical learning. The didactic objectives set for the first and second childhood aim at strengthening the skeletal system and apparatus; direct flexibility training towards the maximum joint excursion; strengthen the cardiovascular system; structure and consolidate the motor and postural patterns specific to gymnastics (rolls, static and dynamic inverted standing position, postural attitudes in suspension, support, balance and in flight) and extend the generic learning to the tools of the respective sections.

Children of gymnastics group recruited for our study carried out 2/3 training sessions per week lasting 1h.30’ up to 2h divided as follows:

Warm-up: jogging and running 12’

- Walk of a technic run
- Quadrupedal walking
- Stretching exercises and joint mobility
- General muscle power
- Handstand care

Technical section for artistic gymnastic equipment:

- Floor exercise, body free 20’ (for all)
- Second equipment 20’
- Third equipment 20’
- Vault 15’ (for all)
- Stretching exercises and joint mobility

*Swimming group training*

The Federal Swimming School supports the fundamental assumption that human beings are not at all suitable to move in water. In the water, there is no maturation and initiation of basic motor skills, which means that any aquatic motor skills must be learned. The swimming school program for second level establishes: 50 m freestyle without interruption, 50 m back style without interruption, 50 m breaststroke without interruption, starting dive, 150 m. Continuous swim without interruption (any technique); knowing how to perform a front flip and a back flip; know how to dive in minimal depth or swim for a short distance in apnea (minimum 12,5 m).

Children of swimming group recruited for our study carried out 2/3 training sessions per week lasting 1h.30’ divided as follows:

Warm-up: only in the water, pre-swimming gymnastics have been abolished since the Covid-19 period and replaced with 10’ of mixed styles at mid speed. Thereafter he followed the technique of one style at time.

- Free style: 50m per 16 times
- Free style: 100m per 8 times with semi-complete rests to initiate the athletes to manage the stopwatch.
- Technique: exercises to improve the technique of the arms and legs in the different styles: both separately (arms only with fin, or legs only with board) and simultaneously, varying: speed, amplitude, direction.

*Athletics group training*

The learning and maturation of motor patterns determines the enrichment of the motor literacy and creates good foundations for preparatory activities for athletics. With this statement, the Italian Athletics Federation establishes the basic principle of youth training. At 6 years old should be proposed basic exercises and short circuits or ball games, then gradually the training becomes more targeted. However, there is time to specialize, this usually occurs when athletes enter the Cadets category (at 14-15 years old) a phase in which they can try their hand at multiple decathlon or heptathlon tests that involve tasks in different specialties of athletic. The competitions start from the beginner categories which are divided into 3 groups: C (6-7 years); B (8-9- years); A (10-11 years) at these ages the competition disciplines are: cross country running: 50 m, 40 m with hurdles, 600 m. foot race, relay race (4x50m and 5x80m), hight and long jump and vortex throw. Then category boys (12-13 years): running races are held at ever greater distances. Competition begins at 14 when young enter the Cadets category.

Children of athletic group recruited for our study carried out 2/3 training sessions per week lasting 1h.30’ divided as follows:

Warm-up: 400m, or 800 m mid speed

- Floor exercise, body free: dynamic exercises and joint mobility. Active starting from the upper body and gradually bust, pelvis, hips, legs, ankles and foots.
- Pre- athletics way of walking, running, leaping (for all)
- Variations of rhythm
- Speed variation

Run increasing the speed

Specialty technical part:

- 20’ runs and/or mixed course which include reactivity exercises with the use of overs and small hurdles, rope, weighted ball, proprioceptive exercises (for all)
- Second specialty 10’ (only for half of the group)
- Third specialty 10’ (only for half of the group).

Inversion of the specialties of the two groups. Final, cool-down run, 400m of athletic track. Stretching.

*Cycling group training*

Finally, the Federal Cycling Regulation provides that athletes can register for a maximum of 28 endurance races (on the road or XCO, Cross Country). Participation in a subsequent endurance race must be carried out at least after 5 days. The road cycling race is considered an endurance race. In this competition there are the most races on the calendar. The volumes of km traveled for the Junior Category (10 years) provides for a race of a maximum 10 km, maximum total of 240 km, based on 24 competitions, 280 km, based on 28.

Children of cycling group recruited for our study carried out 2/3 training sessions per week lasting 1h.30’ of road cycling (closed circuit, for a total of 10 km) divided as follows:

Warm-up:

- 5-10’ muscle activation on the cycle
- 10’ sustained pace along the way, based on their perception of the effort

On an 800mt. circuit:

- 1 lap at high intensity and the next at low intensity
- 5’ of sprint, standing start simulation
- 5’ rest, cool down, mid pace
- 30’ of skills tests: gymkhana, various bike games, obstacle courses.
